# Impact of family communication on the subjective well-being in elderly patients with chronic diseases: A national cross-sectional study

**DOI:** 10.1097/MD.0000000000046739

**Published:** 2026-01-09

**Authors:** Niuniu Sun, Yu Xing, Yifan Yin, Yongqiang Gao, Yi Hu, Yang Ni, Ruijin Zhu

**Affiliations:** a School of Nursing, Henan University of Science and Technology, Luoyang, Henan, China; b School of Public Health, Qingdao University, Qingdao, Shandong, China; c School of Nursing, Shandong Second Medical University, Weifang, Shandong, China.

**Keywords:** chronic diseases, family communication, health literacy, older adults, self-efficacy, subjective well-being

## Abstract

Subjective well-being is a key indicator of healthy aging. However, its relationship with family communication still requires thorough discussion. Data from the 2022 Psychology and Behaviour Investigation of Chinese Residents survey was utilized. The World Health Organization-5 Well-being Index assessed subjective well-being. The Brief Health Literacy Scale, the 10-Item Family Communication Scale, and the Brief Self-Efficacy Scale measured health literacy, family communication, and self-efficacy, respectively. A structural equation model verified path relationships. This cross-sectional study involved 2201 elderly chronic disease patients aged 60 and above. Initially, multiple collinearity tests and common method analysis were conducted, followed by determination of covariates through partial correlation analysis. After controlling for covariates, the results of the structural equation model showed a good fit for the sequential mediation model, with all paths being significant. The subjective well-being of the elderly chronic disease patients is positively correlated with family communication. Health literacy and self-efficacy play a chain mediating role in this relationship.

## 1. Introduction

Currently, there is a steady increase in the aging of the global population. According to the 2020 Census, 18.7% of the population will be over the age of 60^[1]^ increasing the current burden on families and public health systems.^[2]^ The aging of the population has led to an increase in the prevalence of chronic diseases, which are increasing worldwide and result in approximately 41 million (71%) deaths annually.^[3]^ In China, chronic diseases have become the leading cause of death, especially among the elderly population.^[4]^ As aging is associated with a decline in multi-organ reserve function and an increase in the prevalence of multiple chronic diseases, older adults may face challenges in maintaining physical health and quality of life.^[5]^ Moreover, as the country with the largest elderly population,^[6]^ China deserves more attention on how to achieve healthy aging.

Subjective well-being (SWB) is an important indicator of successful aging in a country or region^[7]^; it improves the quality of life and reduces the mortality rate of older adults.^[8,9]^ SWB refers to various subjective assessments of one’s life, including cognitive assessments and emotional feelings.^[10]^ The cognitive component refers to life satisfaction, which is a judgmental process in which people evaluate their quality of life based on individually unique criteria.^[11]^ The affective component refers to well-being, which is an emotional assessment of the intensity of happy moments and the content of positive personal experiences in one’s life.^[11]^ Numerous studies have shown that better mental health can prevent the onset of disease.^[12]^ For example, higher SWB is positively associated with lower rates of cancer, type 2 diabetes, depression, and cardiovascular disease.^[13,14]^ Individuals with chronic diseases are often associated with lower SWB due to long-term medication, dietary control, and pain associated with the disease.^[15]^ The outline of the “Healthy China 2030” plan proposes to identify and intervene in psychological problems in key groups such as chronically ill older adults. Therefore, it is necessary to extensively explore the factors and potential pathways affecting SWB.

Family communication is the act of sharing ideas, participating in decision-making, and expressing feelings among members as a family unit.^[16]^ Communication plays an important role in the functioning of any family, which affects the mental health and well-being of each family member, and to some extent can improve the self-management behaviors of elderly patients with chronic diseases.^[17]^ Self-management of chronic diseases is a long-term process that requires certain knowledge and skills as well as family support and can be acquired through different ways of education and resource support.^[18]^ Health literacy, as an ability to search for health information, helps the elderly chronic disease population to obtain the necessary health information, while the elderly people’s ability to search for health information on their own is weak under the role of this information age, and the support and assistance of family members can facilitate the dissemination of information, thus enhancing the health literacy of elderly chronic disease patients. When patients have adequate health literacy, they are more likely to detect signs of illness, seek treatment, and the psychological burden caused by illness may be reduced.^[19]^ Additionally, family support can increase the self-efficacy of older adults in the community, directly or indirectly influencing healthy aging through health-promoting behaviors.^[3]^ However, there is a lack of certain pathways to analyze how family communication affects the SWB of people with chronic diseases.

In summary, the purpose of this study was to investigate the relationship between SWB and family communication, self-efficacy, and health literacy among Chinese elderly patients with chronic diseases. In addition, it aimed to investigate the mediating role of self-efficacy and health literacy in the relationship between family communication and SWB in Chinese elderly patients with chronic diseases.

### 1.1. Theory and hypotheses

#### 1.1.1. Relationship between family communication and SWB

Positive family communication, including aspects of listening, talking, self-disclosure, clarity, continuity tracking, and respect, may improve physical and mental health through social support and adaptive coping strategies for stressors.^[16]^ Research has shown that family communication has a significant impact on SWB.^[20]^ Effective family communication can help patients understand the trauma caused by the disease, provide them with good family emotional support, and reduce loneliness and other emotional distress to effectively resist the negative events they experience.^[3]^ Therefore, effective family communication is associated with higher SWB in older patients with chronic diseases, and we proposed the research hypothesis H1.

H1: There is a significant positive correlation between family communication and SWB.

#### 1.1.2. Mediating effects of health literacy

Health literacy is an individual’s ability to access, process, and understand basic health information and services needed to make appropriate health decisions: basic functioning (i.e., literacy and numeracy), interactivity (i.e., a patient’s willingness to enter into a co-establishment partnership with a healthcare provider), and critical skills (i.e., a patient’s ability to distinguish between available health services).^[3]^ Research has found that health literacy is positively associated with family communication.^[21]^ In this age of information technology, family members are the main source of health information for older adults, and they utilize various media channels to obtain effective health information and provide health information to the older adult population through communication and other means.^[22]^ Well-communicating families tend to share health-related information more freely and are able to communicate openly and effectively about health issues, helping to create an environment in which health literacy develops and personal health-related knowledge, attitudes, and behaviors are formed.^[3]^

In addition, by investigating the relationship between health literacy and SWB, a positive correlation was found.^[23]^ Health literacy affects a person’s health behaviors, which in turn affects health outcomes^[24]^; it is essential for reducing people’s mental health problems.^[25]^ People with adequate health literacy can learn how to promote individual health by searching for disease-related knowledge, thereby reducing unpleasant experiences caused by disease, such as anxiety, depression, and other psychological problems.^[15]^ Based on the above, we proposed the second hypothesis of this study.

H2: Health literacy mediates the relationship between family communication and SWB.

#### 1.1.3. Mediating effects of self-efficacy

Self-efficacy is the belief in a person’s ability to plan and execute a plan of action, which can be conceptualized as either general or specific, with general self-efficacy referring to confidence in managing actions in a variety of situations.^[26]^ According to Bandura social cognitive theory, individuals acquire or develop self-efficacy through 4 sources of information, one of which includes verbal communication.^[27]^ A longitudinal study suggests that communication between family members can contribute to self-efficacy, for example a serious illness diagnosis may prompt patients to express their emotions and needs to their family members, which over time can influence their self-efficacy and increase confidence in overcoming their illness.^[28]^

Self-efficacy plays an important role in the elderly population as a positive resource in the fight against illness. Self-efficacy enhances subjective well-being in older adults; at the psychological level, older adults’ emotion regulation is influenced by self-efficacy, and positive emotions increase their mental toughness and enhance their well-being, and at the physical level, self-efficacy promotes motivation, which contributes to a sense of SWB.^[29]^ This suggests that the enhancement of SWB is influenced by self-efficacy and that family communication influences the acquisition of self-efficacy. Therefore, we proposed the third hypothesis of this study.

H3: Self-efficacy mediates the relationship between family communication and SWB.

#### 1.1.4. Chain mediating effects of self-efficacy and health literacy

The health literacy skills conceptual framework hypothesizes a relationship between health literacy and health-related outcomes and illustrates how health literacy works at the individual level, while recognizing that factors external to the individual (e.g., family, environment, community, culture, and media) can influence all of the relationships represented in the framework.^[30]^ This suggests that certain health-related stimuli are required for the development of health literacy competencies, where interpersonal communication (including family members, physicians, or friends) is critical to an individual’s skill in interpreting the information conveyed, and self-efficacy may act as a mediator between health literacy and health outcomes.^[30]^ Therefore, family members can help older adults overcome perceived ageism through communication to increase their self-efficacy and use multiple ways to seek scientific health information to improve health literacy, thereby reducing psychological distress associated with illness.^[3]^ Based on the above, we proposed a 4th hypothesis.

**Figure 1.**
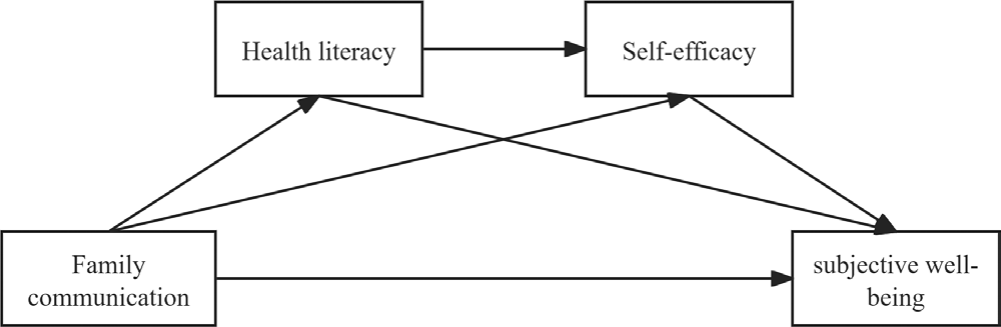
Hypothesis diagram of mediating model between health literacy and self-efficacy in family communication and well-being.

H4: Health literacy and self-efficacy may mediate the sequential mediation effect of family communication on SWB among elderly chronic disease patients (see Fig. 1 for the hypothesis diagram of the mediation model).

## 2. Methods and measures

### 2.1. Participants

This study employed a cross-sectional design based on the data from the 2022 Psychology and Behavior Investigation of Chinese Residents (PBICR). The PBCIR is a nationally representative survey covering 148 cities in 23 provinces, 5 autonomous regions, and 4 municipalities directly under the central government, as well as 202 districts/counties, 390 townships/streets, and 780 communities/villages. The survey was conducted from June to August 2022. A total of 31,480 questionnaires were distributed for the study, with 30,505 valid responses collected, resulting in an effective response rate of 96.9%.

**Figure 2.**
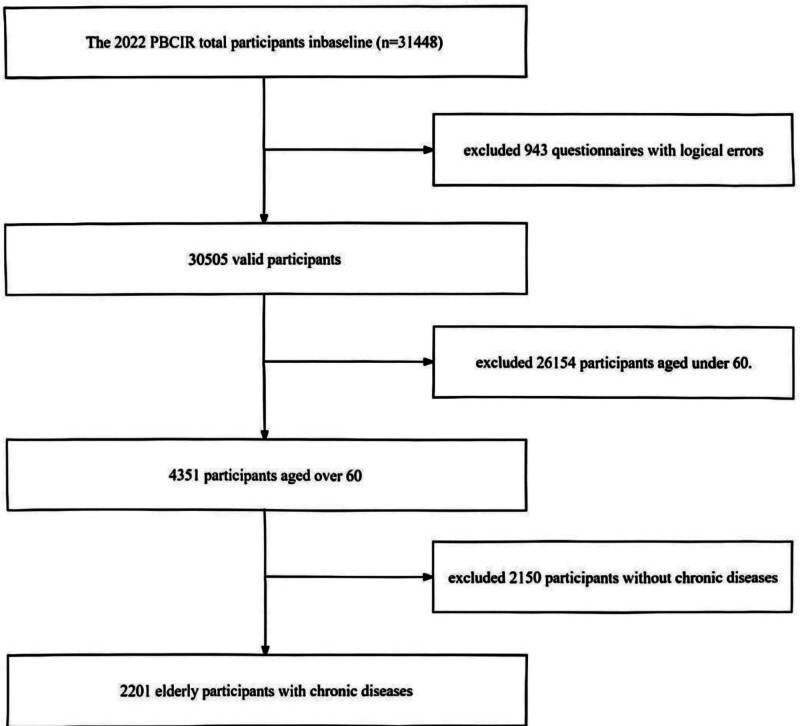
The flowchart of participant selection.

The inclusion criteria for this study were as follows: aged 60 years and above; diagnosed with chronic diseases; permanent residents of China (out-of-town time ≤ 1 month); voluntary participation in the study and signing of informed consent forms; exclusion criteria included having psychiatric disorders, communication barriers, or inability to cooperate with surveyors. Ultimately, 2201 valid questionnaires were collected for analysis. The detailed process of data screening is presented in Figure 2.

### 2.2. Data collection

Each provincial-level administrative region was overseen by a provincial-level coordinator who was responsible for recruiting, training, organizing, and coordinating surveyors/teams within their respective provinces. In each city, at least one surveyor or one survey team was recruited. Each surveyor was accountable for collecting 30 to 90 questionnaires, while each survey team was responsible for gathering 100 to 200 questionnaires. Sampling at the provincial, municipal, district/county, township/street, and community/village levels was carried out through stratified sampling. Meanwhile, sampling from the community/village to the individual level was implemented through non-probability quota sampling. Surveyors set up questionnaire collection points at health service centers or related health service stations in the sampled communities and villages. They put up posters, distributed paper or electronic recruitment notices to enlist participants, and verified the identities of participants to ensure that they met the inclusion criteria and did not meet the exclusion criteria.

### 2.3. Transparency and openness

We report how we determined our sample size and describe all manipulations and measures that were collected. The analysis code can be found on the website https://osf.io/jd8m3/?view_only=1e1165e1aeaf4ef6a9b884422b4a63b5. The data supporting the findings of this study are available from the PBICR. However, the availability of these data is restricted as they were used under license from the PBICR team and cannot be publicly accessed. PBICR will provide access to validate the results of this study upon written request (pbicr_zgjmxlyxwdc@163.com).

### 2.4. Ethical considerations

This study has been approved by the Ethics Committee of the Shaanxi Provincial Health Culture Research Center (JKWH-2022-02) and the Second Xiangya Hospital of Central South University (2022-K050). This study is being registered in the Chinese Clinical Trials Registry (registration number: ChiCTR2200061046).

### 2.5. Methods

#### 2.5.1. Demographic characteristics survey questionnaire

Demographic characteristics survey questionnaire include gender, educational, administrative registration status (rural or urban), marital status (single, married, widowed, divorced), household registration type, average household income, and other general information.

#### 2.5.2. World Health Organization-5 well-being index (WHO-5)

The WHO-5 was first introduced in 1998 by the European Regional Office of the World Health Organization.^[31]^ The scale was translated and revised into Chinese by Ou et al,^[32]^ containing 5 items rated on a scale from 0 to 5,and recommended for measuring the well-being of the elderly. The total score ranges from 0 to 25, with higher scores indicating better mental health. The Cronbach α coefficient for the entire scale in this survey was 0.938.

#### 2.5.3. Health Literacy Scale Short-Form

Health literacy was measured using the internationally adapted abbreviated version of the Health Literacy Scale Short-Form, developed by scholars including Sun Xiaonan.^[33]^ This scale comprises 9 items, with scores ranging from 9 to 36 points. Higher scores indicate higher levels of health literacy. The scale encompasses 3 dimensions: healthcare (3 items), health promotion (3 items), and disease prevention (3 items). Respondents rated each item using a Likert 4-point scale, ranging from 1 to 4, indicating “very difficult” to “very easy.” The overall Cronbach α coefficient for this survey was 0.918, indicating good internal consistency. Confirmatory factor analysis yielded the following results: *x*^2^/df = 5.536, root mean square error approximation degree (RMSEA) = 0.045, adjusted goodness of fit index = 0.976, Tucker-Lewis index (TLI) = 0.985, comparative fit index (CFI) = 0.991, incremental fit index = 0.991, goodness-of-fit index = 0.988. These results demonstrate that the scale developed for this study possesses satisfactory reliability and validity.

#### 2.5.4. The Family Communication Scale-10

Family communication was assessed using the Family Communication Scale-10 developed by Olson et al.^[34]^ This scale comprises ten items, each offering 5 response options ranging from “strongly disagree” to “strongly agree.” Scores range from 10 to 50, with higher scores indicating higher quality of family communication. The overall Cronbach α coefficient for the scale in this study was 0.956, indicating excellent internal consistency.

#### 2.5.5. The New General Self-efficacy Scale

New General Self-efficacy Scale, developed by Chen^[35]^ and revised by Feng,^[36]^ consists of 3 items after being streamlined by the PBICR research group. It employs a Likert 5-point scoring system, ranging from 1 (strongly disagree) to 5 (strongly agree). The total score ranges from 3 to 15, with higher scores indicating higher levels of self-efficacy. The overall reliability and validity of the scale in this study were assessed with a Cronbach α coefficient of 0.914, average variance extracted = 0.7829, and composite reliability = 0.9153. The results indicate good reliability and validity of the scale developed for this research.

#### 2.5.6. Statistical methods and analysis ideas

After data screening and cleaning, we will import the data into SPSS 26.0 (IBM SPSS Statistics, Armonk) for analysis. For descriptive statistics, count data will be described using frequency (n) and percentage (%), while the 4 study variables, which approximate a normal distribution, will be represented using mean (*x*) and standard deviation (*s*). Regarding the differences in the subject well-being across different demographic characteristics, one-way analysis of variance will be employed if the assumption of homogeneity of variance is met. If the assumption is violated, Welch test will be used.

Factors with statistical significance from the one-way analysis results will be included as control variables in partial correlation analysis models to explore the relationships between variables. Subsequently, Mplus 8.3 software (Muthen & Muthen, Los Angeles) will be utilized to conduct bootstrap mediation analysis to examine chain mediation effects, using factors with statistical significance from the one-way analysis as control variables. The significance level (α) will be set at 0.05, and the data will be verified to meet the conditions for the chosen methods.

## 3. Results

### 3.1. Multicollinearity and common method bias test

Multicollinearity tests were conducted on the 4 variables: family communication, health literacy, self-efficacy, and SWB. The results showed that all predictor variables had variance inflation factors below 2.0, indicating the absence of multicollinearity issues.

To test for common method bias, the controlling for the effects of an unmeasured latent methods factor was employed. The results in Table 1 indicate that after adding the global factor (model 2), the changes in CFI and TLI were within 0.1, and the changes in RMSEA and standardized root mean square residual (SRMR) were within 0.05. This suggests the absence of significant common method bias.^[37]^

**Table 1:** The result of unmeasured latent method construct.

	RMSEA	CFI	TLI	SRMR
Model 1	0.052	0.961	0.957	0.027
Model 2	0.054	0.958	0.953	0.039

*Note*: model 1 represents the CFA results of the four-factor model, and model 2 represents the CFA results after adding the global factor.

CFI = comparative fit index, RMSEA = root mean square error approximation degree, SRMR = standardized root mean square residual, TLI = Tucker-Lewis index.

### 3.2. Univariate analysis of sociodemographic factors

The mean age and standard deviation of all participants (N = 2201) were 69.73 ± 6.56 years old, and 50.9% participants were male. 49.9% participants had an educational level of primary school or below, 80.7% were married, and 45.6% had a family annual income of <3000 China Yuan. Additionally, 83.8% of participants suffered from 1 to 2 chronic diseases, and 44.2% resided in rural areas. Furthermore, 14.6% of elderly chronic disease patients lived alone. Significant differences were found in SWB scores concerning age, educational level, marital status, number of chronic diseases, residence, and living alone status. For more detailed information, please refer to Table 2.

**Table 2:** Demographic characteristics of elderly patients with chronic disease.

Variable	*N* (%)	WHO-5
Gender		
Male	1121 (50.9)	13.91 ± 5.71
Female	1080 (49.1)	14.19 ± 5.59
* F/Welch*		1.332
* P*		.248
Age		
60–69	1088 (49.4)	14.43 ± 5.77
≥70	1113 (50.6)	13.67 ± 5.51
* F/Welch*		10.009
* P*		.002
Education		
Primary school and below	1099(49.9)	13.14 ± 5.24
Junior high school	455(20.7)	14.49 ± 5.88
Senior high school	362(16.4)	14.79 ± 5.97
College and beyond	285(12.9)	15.93 ± 5.77
* F/Welch*		23.648
* P*		<.001
Marital status		
Single, divorced or widowed	425 (19.3)	13.39 ± 5.42
Married	1776 (80.7)	14.21 ± 5.70
* F/Welch*		7.290
* P*		.007
Household monthly income (CNY)		
≤3000	1003 (45.6)	13.83 ± 5.26
3001–6000	894 (40.6)	14.33 ± 6.05
6001–9000	179 (8.1)	13.70 ± 5.19
≥9001	125 (5.7)	14.36 ± 6.35
* F/Welch*		1.543
* P*		.203
Number of chronic diseases		
1–2	1844 (83.8)	14.17 ± 5.66
≥3	357 (16.2)	13.54 ± 5.41
* F/Welch*		5.300
* P*		.021
Residence		
Rural	972 (44.2)	12.91 ± 5.37
Urban	1229 (55.8)	14.95 ± 5.71
* F/Welch*		72.914
* P*		<.001
Living alone		
Yes	321 (14.6)	13.3 ± 5.45
No	1880 (85.4)	14.18 ± 5.68
* F/Welch*		6.593
* P*		.010

CNY = China Yuan.

### 3.3. Partial correlation analysis of the main variables

We conducted a partial correlation analysis on the primary outcome variables, controlling for statistically significant factors identified in the single-factor analysis of variance as covariates. The results indicated that the average score for family communication was 36.60 ± 7.80. The average score for self-efficacy was 10.37 ± 2.45, and for health literacy, it was 24.58 ± 5.38. Additionally, the average score for SWB was 14.05 ± 5.65. After controlling for age, education, marital status, number of chronic diseases, residential area, and living alone status, family communication showed significant positive correlations with health literacy (*R* = 0.580, *P* < .001), self-efficacy (*R* = 0.567, *P* < .001), and SWB (*R* = 0.472, *P* < .001), with moderate effect sizes. Health literacy was significantly positively correlated with self-efficacy (*R* = 0.435, *P* < .001) and SWB (*R* = 0.391, *P* < .001), also exhibiting moderate effect sizes. Self-efficacy showed a significant positive correlation with SWB (*R* = 0.587, *P* < .001), with moderate effect sizes. For more detailed information, please refer to Table 3.

**Table 3:** Partial correlation coefficients of major variables.

Variables	Mean	SD	Family communication	Health literacy	Self-efficacy	Subject well-being
Family communication	36.60	7.80	1			
Health literacy	24.58	5.38	0.580**	1		
Self-efficacy	10.37	2.45	0.567**	0.435**	1	
Subject well-being	14.05	5.65	0.472**	0.391**	0.587**	1

*Note*: All tests were two-tailed. Means and standard deviations (SD) are presented for each variable. Correlation coefficients represent partial correlations controlling for other variables.

** *P* < .01.

### 3.4. Pathway testing of the structural equation models

When confronted with an extensive questionnaire, employing the original items directly in modeling may lead to significant parameter estimation bias.^[38]^ The item parceling approach was used for modeling. For the family communication scale, which consisted of a single dimension, the method of item-to-construct balance proposed by Rogers et al^[39]^ was employed to create 5 indicators. For the health literacy scale with 3 dimensions, isolated parceling was used to aggregate each dimension into one indicator for analysis.^[40]^

Previous studies have identified that the determinants of SWB for elderly included individual, societal, and environmental factors.^[41]^ In order to prevent these demographic characteristics from affecting the relationship between family communication and SWB and ensure the accuracy of the results, they were used as control variables in mediation and moderation analysis. Controlling for factors with statistically significant results in the univariate analysis, we constructed a structural equation model. According to the recommendations of Hu and Bentler,^[42]^ a model fit was considered good if it met certain criteria, including RMSEA ≤ 0.06, CFI and TLI ≥ 0.95, and SRMR ≤ 0.08. The current model indices were as follows: CFI: 0.969, TLI: 0.965, RMSEA: 0.050, SRMR: 0.079, indicating a good model fit.

**Figure 3.**
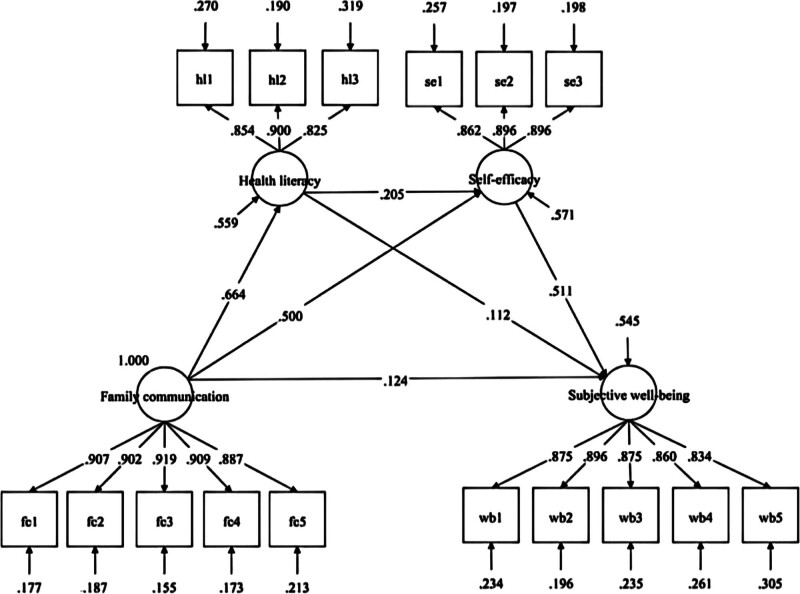
The path analysis of the structural equation model. *Note*: All path coefficients were standardized.

Figure 3 illustrate the results of the path analysis of the structural equation model. Family communication positively predicted health literacy (*β* = 0.664, *P* < .001) and self-efficacy (*β* = 0.500, *P* < .001). Additionally, there was a statistically significant direct effect of family communication on SWB (*β* = 0.124, *P* < .001). Apart from the relatively small effect size on SWB, the effects of family communication on self-efficacy and health literacy were both moderate. Health literacy positively predicted self-efficacy (*β* = 0.205, *P* < .001) and SWB (*β* = 0.112, *P* < .001), with relatively small effect sizes for both paths. Similarly, self-efficacy positively predicted SWB, with a large effect size (*β* = 0.511, *P* < .001).

### 3.5. Tests of direct and indirect effects in the sequential mediation model

We used the Bootstrap method to analyze the mediating effect between family communication and SWB, with an iteration of 2000 and a confidence interval of 95%. If the 95% confidence interval did not include 0, it indicated a significant mediating effect. The results of the mediation analysis showed that health literacy and self-efficacy mediate the relationship between family communication and SWB, with a total mediation effect of 0.524. There were 3 mediation pathways: family communication → health literacy → SWB (0.097), family communication → self-efficacy → SWB (0.335), and family communication → health literacy → self-efficacy → SWB (0.091). The 95% confidence intervals for all paths did not include 0, indicating significant mediation effects. The proportions of mediation effects were 14.14%, 48.83%, and 13.27% for 3 pathway, respectively, with the total mediation effect accounting for 76.38% of the total effect. All hypothesized paths were supported. For more detailed information, please refer to Table 4.

**Table 4:** Tests of direct and indirect effects in the sequential mediation model.

	Effect	BootSE	BootLLCI	BootULCI	Relative mediation effect
Total effect	0.686	0.030	0.641	0.738	
Total indirect effect	0.524	0.034	0.467	0.580	76.38%
Family communication → health literacy → SWB	0.097	0.028	0.051	0.143	14.14%
Family communication → self-efficacy → SWB	0.335	0.026	0.296	0.379	48.83%
Family communication → health literacy → self-efficacy → SWB	0.091	0.014	0.069	0.116	13.27%

*Note*: All variables in the model have been standardized.

## 4. Discussion

SWB is crucial for elderly people, as those with higher SWB tend to live longer and have healthier lives compared to those with lower SWB.^[43]^ Elderly people with chronic diseases often experience lower SWB compared to those without chronic conditions.^[13]^ It is worthwhile to explore the influencing factors of SWB among elderly people with chronic diseases. This study surveyed 2201 elderly people with chronic diseases in China, and the results showed that family communication was positively related to SWB, and health literacy and self-efficacy partially mediated this relationship. This study not only explored the relationship between family communication and SWB but also extended the current research on influencing factors of SWB. Elderly chronic disease patients with high family communication, health literacy and self-efficacy has a high level of SWB. Therefore, this study can provide theoretical and practical significance for enhancing SWB among elderly people with chronic diseases.

Based on the results of this study, we found that family communication was positively related to SWB of elderly patients with chronic illnesses. Hypothesis H1 is confirmed. This finding is consistent with that of Dai et al,^[44]^ who suggests that the relationships between elderly people and their family members is associated with higher levels of SWB. As the circumplex model suggests, high levels of family communication can promote the formation of a balanced family and lead to better family functioning.^[45]^ Good communication helps family members to change their levels of cohesion and adaptability, and leads the family towards a balanced state.^[46]^ A balanced family can enable family members to better fulfill essential family functions, including economic, psychological, and health-related care, which are particularly important for chronic disease patients.^[47]^ In a family where members communicate actively, individuals can feel understood, supported, and cared for, which helps them experience a higher level of well-being.

This study showed that health literacy has a mediating effect on the relationship between family communication and SWB, supporting H2. Consistent with previous research, elderly chronic disease patients with good family communication has better health literacy.^[21]^ Low health literacy levels are not only associate with chronic disease patients’ insufficient understanding of their health conditions, poor self-care abilities, delayed medical care, and low preventive service utilization rates, but also affect chronic disease management and outcomes, leading to lower SWB.^[48–51]^ The mediating effect of health literacy confirmed the Family Communication Patterns Theory, that is, better family communication leaning towards conversation orientation, and such orientation more likely to enhance the general well-being of individual family members.^[52]^ Better family communication can predict more health communication topics, influence family members’ health behaviors,^[53]^ and families with a high family communication level encourage their members to share their thoughts and knowledge. For example, elderly individuals often face barriers in accessing media information and using new information technologies, leading them to rely on their children’s opinions when identifying health information on digital media,^[54]^ communication with their children can help them understand more disease information, promote their health literacy.^[55]^ Therefore, attention should be given to the health literacy of elderly people with chronic diseases, as improving health literacy can enhance their subjective well-being.

This study also found the mediating role of self-efficacy between family communication and SWB in elderly chronic disease patients hypothesis H3 is supported. Self-efficacy is mainly influenced by 4 factors: performance accomplishments, vicarious experience, verbal persuasion, and emotional arousal.^[56]^ Good family communication not only provides more encouragement, advice, and suggestions but also helps elderly people make medical decisions and supports or provides the necessary care.^[57]^ These behaviors can increase self-efficacy in elderly patients with chronic diseases in the form of performance accomplishments and verbal persuasion. For elderly people with chronic diseases, disease management is crucial and closely related to their sense of happiness.^[13]^ Elderly people may have been living with chronic diseases for many years, and they make daily disease management decisions regarding lifestyle behaviors.^[58]^ Patients with low self-efficacy are more likely to exhibit poor self-management.^[59]^ Future interventions and preventive efforts could targeting the improvement of self-efficacy among the elderly, increasing their ability for self-management and ultimately enhancing their SWB.

The results of this study also confirmed hypothesis H4. The variable of family communication was introduced in this study, and our result confirmed the hypothesis in the health literacy skill framework, that is, health literacy can influence health outcomes (such as subjective well-being) through mediating variables like self-efficacy, and family influence all relations represented in the framework.^[30]^ On one hand, after being diagnosed with chronic illnesses, patients frequently utilize diverse sources of health-related information to formulate health decisions, with familial and peer networks constituting primary conduits.^[60]^ On the other hand, individuals with higher health literacy are more likely to understand information from medical institutions and doctors, master medical skills, adequately prepare for medical visits, streamline medical processes, reduce the costs associated with multiple visits to medical institutions, and engage in shared decision-making.^[61]^ Positive communication between family members can provide the necessary support for these activities. These activities amplify their sense of self-efficacy, encouraging patients to better implement health knowledge in practice, thereby reducing negative impacts of chronic disease. More health-promoting behaviors, which are associated with better self-management of chronic diseases, can enhance emotional regulation, facilitate the return of the body to its normal state,^[25]^ and thereby improve subjective well-being. Therefore, in medical interventions, it is important to not only focus on patients’ family communication but also on enhancing their health literacy and self-efficacy. This can be achieved through education, support, and promoting positive health behaviors, which helps elderly patients with chronic disease achieve better subjective well-being.

## 5. Limitations and future directions

Although this study employed rigorous sampling and a nationally representative sample, there are still some limitations. First and foremost, the cross-sectional design limits the establishment of causal relationships. Future research should employ longitudinal designs to determine the directionality of these variables. Secondly, while the WHO-5 can provide a general assessment of SWB,^[62]^ SWB has different definitions across various cultural backgrounds.^[63]^ Therefore, future research could explore specific domains of SWB. Lastly, the study only focuses on health literacy and self-efficacy as mediating variables, neglecting other potential mechanisms. Future research should explore more social, familial, and individual factors related to SWB.

## 6. Conclusion

This study focuses on elderly people with chronic illnesses and employs structural equation modeling to examine the relationship between family communication and SWB among them. The results reveal the mediating role of health literacy and self-efficacy. These findings are of significant importance for understanding the roles of family communication, self-efficacy, and health literacy in the SWB of elderly patients with chronic illnesses. They provide valuable insights for enhancing the SWB of elderly patients with chronic illnesses, offering useful directions for improvement.

## Acknowledgments

We would like to thank PBICR database for providing research data for this paper, my partner Yin Yifan for his outstanding contributions to this article, and the meticulous guidance of my mentor.

## Author contributions

**Conceptualization:** Yu Xing, Yongqiang Gao, Yang Ni, Ruijin Zhu.

**Data curation:** Yu Xing, Yifan Yin, Yang Ni.

**Formal analysis:** Yongqiang Gao.

**Investigation:** Yi Hu.

**Methodology:** Yifan Yin, Yi Hu.

**Supervision:** Niuniu Sun.

**Writing – review & editing:** Niuniu Sun.

**Writing – original draft:** Yu Xing.
